# Supplementary material for: Change, Adversity, and Adaptation: Young People’s Experience of the COVID-19 Pandemic Expressed through Artwork and Semi-Structured Interviews
Source: Int J Environ Res Public Health. 2024 May 16;21(5):636. doi: 10.3390/ijerph21050636 (PMC11121572; doi:10.3390/ijerph21050636)
Supplement: Supplementary file 1 [file ijerph-21-00636-s001.zip › ijerph-2966085-supplementary.pdf]

Supplementary Materials for Thompson et al.'s "*Change, adversity, and adaptation: young people's experience of the COVID-19 pandemic expressed through artworks and interviews*"

Table S1. Consolidated criteria for reporting qualitative studies (COREQ): 32-item checklist [44]

**No. Item**	**Guide questions/description**	**Reported on Page #**
**Domain 1: Research team and reflexivity**		
Personal Characteristics		
1. Interviewer/facilitator	Which author/s conducted the interview or focus group?	4
2. Credentials	What were the researcher's credentials? E.g. PhD, MD	4
3. Occupation	What was their occupation at the time of the study?	4
4. Gender	Was the researcher male or female?	4
5. Experience and training	What experience or training did the researcher have?	4
Relationship with participants		
6. Relationship established	Was a relationship established prior to study commencement?	4
7. Participant knowledge of the interviewer	What did the participants know about the researcher? e.g. personal goals, reasons for doing the research	4
8. Interviewer characteristics	What characteristics were reported about the interviewer/facilitator? e.g. Bias, assumptions, reasons and interests in the research topic	4
**Domain 2: study design**		
Theoretical framework		
9. Methodological orientation and Theory	What methodological orientation was stated to underpin the study? e.g. grounded theory, discourse analysis, ethnography, phenomenology, content analysis	5
Participant selection		

10. Sampling	How were participants selected? e.g. purposive, convenience, consecutive, snowball	4
11. Method of approach	How were participants approached? e.g. face-to-face, telephone, mail, email	4
12. Sample size	How many participants were in the study?	6
13. Non-participation	How many people refused to participate or dropped out? Reasons?	6
Setting		
14. Setting of data collection	Where was the data collected? e.g. home, clinic, workplace	4-5
15. Presence of non-participants	Was anyone else present besides the participants and researchers?	4
16. Description of sample	What are the important characteristics of the sample? e.g. demographic data, date	6-7
Data collection		
17. Interview guide	Were questions, prompts, guides provided by the authors? Was it pilot tested?	3-5
18. Repeat interviews	Were repeat inter views carried out? If yes, how many?	N/a
19. Audio/visual recording	Did the research use audio or visual recording to collect the data?	5
20. Field notes	Were field notes made during and/or after the inter view or focus group?	Supplementary Information C, p6
21. Duration	What was the duration of the inter views or focus group?	6
22. Data saturation	Was data saturation discussed?	6
23. Transcripts returned	Were transcripts returned to participants for comment and/or correction?	N/a
**Domain 3: analysis and findings**		
Data analysis		
24. Number of data coders	How many data coders coded the data?	5
25. Description of the coding tree	Did authors provide a description of the coding tree?	N/a
26. Derivation of themes	Were themes identified in advance or derived from the data?	5
27. Software	What software, if applicable, was used to manage the data?	**5**
28. Participant checking	Did participants provide feedback on the findings?	N/a

Reporting		
29. Quotations presented	Were participant quotations presented to illustrate the themes/findings? Was each quotation identified? e.g. participant number	8-21
30. Data and findings consistent	Was there consistency between the data presented and the findings?	6-22
31. Clarity of major themes	Were major themes clearly presented in the findings?	6-22
32. Clarity of minor themes	Is there a description of diverse cases or discussion of minor themes?	6-22

Table S2. GRIPP2 short form [45]

Section and topic	Item	Reported on page No
1: Aim	Report the aim of PPI in the study	3
2: Methods	Provide a clear description of the methods used for PPI in the study	4
3: Study results	Outcomes—Report the results of PPI in the study, including both positive and negative outcomes	24-25
4: Discussion and conclusions	Outcomes—Comment on the extent to which PPI influenced the study overall. Describe positive and negative effects	24-26
5: Reflections/critical perspective	Comment critically on the study, reflecting on the things that went well and those that did not, so others can learn from this experience	24-26

PPI=patient and public involvement

Table S3. COVID Qualitative sub-study: topic guide

Pre-interview

- **Initially –** join the teams meeting or call number provided. File recording should be titled "*ID number*_interview_*date").*
- Welcome: Remind them that the purpose of interview is to understand their experience of the COVID-19 pandemic and associated lockdowns. Remind them there is no wrong or right answer, that they are the expert in their own experience, and we are trying to understand their specific experience. We are here to listen and are not judging.
- Remind them of confidentiality and the voluntary nature of their consent
  - Caveat: "if you indicate that you or someone else is at risk, I will have to take this further/ escalate it "
- Explain they can stop the interview at any time. If they feel uncomfortable answering the specific question, they are not pressured to answer It and can 'pass'.
- Give summary of Interview structure, including number of questions and topics
- Ask if they have any questions.
- Inform that recording will now begin and start recording.

<u>Interview</u>
Questions
Numbered = Always asked
- = Asked if elaboration/ extra detail is required

The order of 8 and 9 will depend on the individual and flow of conversation.

1. How did you find the workshop?
2. Please tell me about what you created in the workshop.
- Possible follow-up for explanation, e.g. ‘tell me about this element’, ‘how come you chose X’
- What do you think your work says about COVID? What did you think other people’s work said about COVID?
3. How did you feel when the first lockdown was announced? What changed for you at this time? Were you worried about the impact it might have on a previous diagnosis or problem from before?
- How did this effect you? (if not elaborated on)
4. What was a good experience you had over lockdown? Has lockdown allowed you to try something new or have extra time for something you enjoy?
5. How did you find learning over the lockdown? How has the support been from school? How has your experience been different compared to people who didn’t go to school during COVID?
- How did you cope with exams and homework during home learning? How many hours during a typical day did home learning happen? Did it tend to be live lessons or independent working? How did this effect your learning or wellbeing in general?
6. How did you keep occupied during lockdown? Were any of your hobbies and pastimes effected by lockdown? Have your hobbies helped you during the pandemic and how?
- What are they?
7. How have your relationships with friends and family been? How did you find communicating with friends and family over lockdown? How did you find the shifts in your day-to-day social life and connections, e.g. at school?
- Have they been impacted, strained, strengthened?
8. Did you find the first and second lockdowns to be similar or different?
- What are the differences? Why might that be?
9. How often did you exercise during the pandemic/lockdown and what sort of exercise did you tend to do? Were your sleeping and eating habits impacted?
- Would you say you became healthier or less healthy or about the same and in what way? Was exercise consistent? Did how much you exercise change as a result of the pandemic/lockdown?
10. Have you experienced any loss during the pandemic? Did you notice any changes to your mental health during these events, positive or negative? How were your anxiety levels and worry? How have you felt throughout lockdown? Did anything particularly help or make you feel worse? How was your access to support?
- Was there any particular situation that especially affected you? Did anything particularly stress you out or sorry you? Did you have enough support?
11. How have you found the lifting of restrictions? How have you re-adapted to face-to-face learning and going back to school?
12. How do you envisage the future? Are you hopeful for the future? Have your expectations or ambitions changed over lockdown?
13. Is there anything which you have gained from this past year? Has how you feel about the pandemic and lockdown itself changed? How do you feel your overall experience of the pandemic and lockdown was?

- How has your outlook changed in the past year? Has the pandemic impacted your perspective on anything?

14. Do you have anything you would like to add?

Extra Questions/ improvising

- Cover all bases-but should feel like a natural conversation, you can rearrange the order if things come up that a later question more naturally follows from.
- You can ask follow-up questions or for clarification, you can explore/dig deeper on something interesting.
- If asking follow-up question: Try and use open, non-directional questions, rather than closed directional questions, and use clear vocabulary.
- Reflections can be useful for clarification, e.g. "it sounds like..."

Body language and conduct

- Nodding, use of non-verbal sounds
- Be engaged, listen, focus on them, don't make notes or look at your phone.
- One question at a time, don't put them together or follow-up too quickly
- Give them time to think and respond, be comfortable with silence (count to 10 in your head before prompting further with another question)
- Don't interrupt
- Speak softly and calmly
- Some people will not say a lot and that's fine, allow silences

Other things

- To push along, reflecting can work quite well-, e.g. "so it sounds like x… "
- Let them know whenever you're recording.
- If someone is distressed, remind them they can stop at any time, skip a question, etc.
- Every interview doesn't need to be the same, they will naturally vary, and some people might be suited to different approaches (e.g. volume, general chitchat, formality, asking you questions)
- If you're asked a question, you don't have to answer (you can say something like 'well, this session isn't about me'). You can answer, but it shouldn't be at length and the conversation should be directed back to them.
- Don't think you know how the person should think, feel or behave. Everyone's response is different.
- Don't use general 'positive' phrases such as 'look on the bright side' or 'look for the silver lining'.
- Don't judge their thoughts or feelings – being accepted helps put things in context.
    - 'Why' questions can feel interrogative, better to use phrases like 'how come'
    - If content has been heavy or distressing, a debrief with other staff is a good idea.

<u>**Post interview**</u>

- Inform participant the recording is ending.
- Ask if they have any questions.
- Explain the research question and objectives (to look at young people's experience of lockdown, particularly regarding their mental health and understanding factors behind why some found it easier or more difficult)
- Aftercare and decompress (see below)

- Provide with handout of support and voucher for their time (you can say these are coming via email).
- Thank them again.
- Save recording with filename: *ID number*_interview_*date*
- File exchange drop to database manager- once picked it up, delete it.

- Please make notes after the interview on anything that was interesting and came up that wasn't captured in recorded responses. For example, anything that would be relevant to evaluating the methodology or for people conducting such research again, or reflexive observations about how your thoughts, feelings and positionality may have influenced the interview.

Aftercare and decompress (Optional, e.g. if participant seems distressed)

- Allow 10 minutes to cool down.
- Thank participant for being so open and honest.
- Ask how they're feeling now.
  - **Empathise** with their feelings. E.g., if they struggled during lockdown, you could say that it sounded like it was difficult and thank you for sharing.
  - **Normalise** their feelings. E.g. If it has come up during the interview that lockdown has been difficult, reassure them that it has been a very difficult time, you could say that lots of people have felt like this, that's why we're doing the research and that their reactions and feelings are normal.
  - **Validate** their feelings. E.g. If it has come up during the interview that lockdown has been difficult you could say "It's completely understandable that talking about this would make you feel xxx"
  - [We will note to ourselves whether the participant needed that time, and make notes personally on what content was covered]

Calming techniques: There are some strategies for calm provided in the hand-out given to participants, but if signs of high distress are apparent during the interview, the following techniques can be used:

- Deep breathing: breathe in for 7 out for 11. When you're upset and stressed your body goes into fight, flight or freeze mode. Taking deep breaths instead of shallow breaths is one way to calm your body down.
- 54321 technique: ask them to notice five things they can see, four things they can touch, three things they can hear, two things they can smell, and one thing they can taste.

Escalating Process:

- If someone (the participant, or someone else they've talked about) is in present danger of harm or abuse.
- Contact local safeguarding lead; [*DETAILS REMOVED FOR PUBLICATION.]*
- Case by case – if there's something we're not sure about, we will check in after each round of interviews and discuss (anonymously) to decide whether to escalate.

References

44. Booth, A.; Hannes, K.; Harden, A.; Noyes, J.; Harris, J.; Tong, A. COREQ (consolidated criteria for reporting qualitative studies). *Guidel. Report. Health Res. User's Man.* **2014**, 214-226.
45. Staniszewska, S.; Brett, J.; Simera, I.; Seers, K.; Mockford, C.; Goodlad, S.; Altman, D.G.; Moher, D., Barber, R.; Denegri, S.; Entwistle, A. GRIPP2 reporting checklists: tools to improve reporting of patient and public involvement in research. *Bmj* **2017** *358*, j3453
